# The Effect of Nrf2 Pathway Activation on Human Pancreatic Islet Cells

**DOI:** 10.1371/journal.pone.0131012

**Published:** 2015-06-25

**Authors:** Yuichi Masuda, Nosratola D. Vaziri, Shiri Li, Aimee Le, Mohammad Hajighasemi-Ossareh, Lourdes Robles, Clarence E. Foster, Michael J. Stamos, Ismail Al-Abodullah, Camillo Ricordi, Hirohito Ichii

**Affiliations:** 1 Department of Surgery, University of California Irvine, Irvine, California, United States of America; 2 Medicine, University of California Irvine, Irvine, California, United States of America; 3 Southern California Islet Cell Resources Center, Department of Diabetes, Endocrinology and Metabolism, City of Hope National Medical Center and Beckman Research Institute, Duarte, California, United States of America; 4 Diabetes Research Institute, University of Miami, Miami, Florida, United States of America; Niigata University School of Medicine, JAPAN

## Abstract

**Background:**

Pancreatic islets are known to contain low level of antioxidants that renders them vulnerable to oxidative stress. Nrf2 is the master regulator of numerous genes, encoding antioxidant, detoxifying, and cytoprotective molecules. Activation of Nrf2 pathway induces up-regulation of numerous genes encoding antioxidant and phase II detoxifying enzymes and related proteins. However, little is known regarding the role of this pathway in human islet cells. The aim was to investigate the effect of Nrf2 activator (dh404, CDDO-9,11-dihydro-trifluoroethyl amide) on human islet cells.

**Methods:**

Human islets were obtained from cadaveric donors. After dh404 treatment, Nrf2 translocation, mRNA expression, and protein abundance of its key target gene products were examined. The proportion of dh404-treated or non-treated viable islet beta cells was analyzed using flowcytemetry. The cytoprotective effects against oxidative stress and production of inflammatory mediators, and *in vivo* islet function after transplantation were determined.

**Results:**

Nrf2 nuclear translocation was confirmed by con-focal microscope within 2 hours after treatment, which was associated with a dose-dependent increase in mRNA expression of anti-oxidants, including NQO1, HO-1, and GCLC. Enhanced HO-1 expression in dh404 treated islets was confirmed by Western Blot assay. Islet function after transplantation (2000 IEQ/mouse) to diabetic nude mice was not affected with or without dh404 treatment. After induction of oxidative stress with hydrogen peroxide (200 μM) the proportion of dh404-treated viable islet cells was significantly higher in the dh404-treated than untreated islets (74% vs.57%; P<0.05). Dh404 significantly decreased production of cytokines/chemokines including IL-1β, IL-6, IFN-γ and MCP-1.

**Conclusion:**

Treatment of human pancreatic islets with the potent synthetic Nrf2 activator, dh404, significantly increased expression of the key anti-oxidants enzymes, decreased inflammatory mediators in islets and conferred protection against oxidative stress in beta cells.

## Introduction

Type 1 diabetes mellitus (T1DM) is an autoimmune disease associated with selected genetic HLA alleles, which result in the permanent destruction of β-cells of the pancreatic islets of Langerhans [1]. Previous studies indicate that antigen-specific T-cells mediate the infiltration of inflammatory cells into the pancreas, which leads to the production of inflammatory cytokines, such as interleukin (IL)-1β, tumor necrosis factor-α (TNF- α), and interferon (IFN)-γ [2,3]. IL-1β, either alone or in combination with TNF- α and IFN-γ, causes the production of reactive oxygen species (ROS), which result in beta cell destruction [4]. Pancreatic islets contain very low levels of the antioxidant enzymes [5]. Therefore, they have an innate vulnerability to oxidative stress and inflammation [6].

Nuclear factor erythroid2-related factor1 (Nrf2)-Kelch-like ECH Associated protein1 (Keap1) signaling pathway plays a significant role in protecting the cells against various stresses including endogenous and exogenous oxidants, inflammatory stresses, and chronic exposures to cigarette smoke and other carcinogens [7–13]. The cytoprotective effects of Nrf2 are mediated by transcriptional up-regulation of genes encoding numerous antioxidant, detoxifying and cytoprotective enzymes and related molecules.

It has been recently reported that Nrf2-keap pathway in pancreatic beta cells plays acritical role for the protection form oxidative stress. Yagishita et al. precisely investigated the role of Nrf2-keap pathway using four genetically modified mouse model. [14]. They demonstrated that the activation of Nrf2-keap1 pathway in islets have advantages against oxidative stress induced by iNOS, on both islet morphology and function. Furthermore, they examined the expression of Nrf2 target genes including NQO1 and HO-1in presence and absence of oxidative stress, and demonstrated the Nrf2-dependent expression of these genes.

Li et al. reported that Nrf2 expression in islets from patients and mice with early stage of diabetes was increased, and that activation of Nrf2 with dh404 (CDDO-9,11-dihydro-trifluoroethyl amide) reduced oxidative stress-induced beta-cell apoptosis while enhancing autophagic clearance in isolated rat islets [15]. We also recently reported the beneficial effects of dh404 on rodent islet isolation model [16]. In Nrf2 knockout mice, the islet yield was significantly decreased. In contrast administration of dh404 in the normal rats increased HO-1 expression and significantly improved islet yield. Furthermore, the reversal rate of diabetes with islet transplantation in diabetic nude mice was significant improved with dh404 treatment.

There is a significant potential for clinical applications of Nrf2 activators in patients with diabetes. However, little data is available regarding the effect of synthetic Nrf2 inducers in human pancreatic islets. The aim of the present study was to test the hypothesis that treatment of human pancreatic islets with a potent synthetic Nrf2 activator (dh404) may improve beta cell viability and function by protection against oxidative stress.

## Materials and Methods

### Reagents

Nrf2 activator, dh404 was provided by Reata Pharmaceuticals, Inc. (Irving, TX). The chemical name for dh404 is CDDO-9,11-dihydro-trifluoroethyl amide (CDDO-dhTFEA) [17].

### Human islets

Human islets were obtained from cadaveric donors through NIH/JDRF sponsored integrated islet distribution program (IIDP, https://iidp.coh.org/). Institutional Review Board exemption was obtained at University of California Irvine. Our study does not involve any information including living individuals, identifiable and private information.

### Immunofluorescence and Confocal Microscopy

Human islets were fixed in 10% formalin and embedded in Optical Cutting Temperature compound, and frozen.10-μm-thick sections were cut, rinsed in phosphate buffered saline (PBS, Corning Manassas, VA), and incubated with Universal Blocker Reagent (Biogenex, San Ramon, CA) for 30 min in a humidified chamber at room temperature. Thereafter, sections were incubated with primary antibodies, rabbit anti-Nrf2 (1:100; AbcamInc, Cambridge, MA) overnight at 4°C in a humidified chamber. After PBS wash, Alexa Fluor 488 antibody (1:100; Molecular Proves, Carlsbad, CA) and a nuclear stain, 4′, 6-diamidino-2-phenylindole (DAPI; Invitrogen) were applied to the slides and incubated at room temperature for 2 h in the dark. The slides were analyzed under the confocal laser scanning microscopy (Zeiss LSM700, Jena Germany), and the images were analyzed by ZEN 2011 (Carl Zeiss).

### Nrf2 target gene analysis in human islets treated with dh404

Human islets from three different donors were cultured and incubated with dh404 at 250 and 1000 nM in Dimethyl Sulphoxide Hybri-MAX (DMSO; 0.1% w/v, Sigma, St. Louis, MO) for 12 hours. Cells were then washed with PBS, pelleted, and stored at -80°C until analysis. Samples were analyzed by the Quantigene Plex 2.0 assay from Panomics (an Affymetrix company) per manufacturer's protocol. Data were normalized to the housekeeping gene POLR2A and presented as fold vehicle. Data were analyzed by one-way ANOVA followed by Duncan’s post-hoc test.

### Western Blot Analysis

Total protein of human islets from 3 donors (both control and treatment group) were obtained with CelLyticNuCLEAR Extraction Kit (Sigma, St. Louis, MO) following the manufactures instruction. In brief, islets were homogenized and lysed on ice with Lysis buffer (including DTT and protease inhibitors) for 20 min. The pancreatic lysate was centrifuged at 11,000 x g for 11 min. The cytoplasmic protein concentration was measured using Bio-Rad DC Protein Assay (Bio-Rad Laboratories, Hercules, CA). The primary antibodies used were HO-1 (1:1000; AbcamInc, Cambridge, MA) and GAPDH (1:5000; Sigma). Aliquots containing 15 μg proteins were loaded on NuPAGE 4–12% Bis-Tris gel (Life Technologies, Grand Island, NY), and then transferred to a PVDF membrane (Pall Life Science, Ann Arbor, MI). The membrane was blocked in TBS-T (Thermo Scientific, Rockford, IL) containing 5% blocking grade non-fat dry milk (Bio-Rad, Hercules, CA), and incubated with primary antibodies overnight at 4°C. After washing, the membrane was further incubated with HRP-conjugated goat anti-rabbit secondary antibody (1:3000; Cell signaling, Danvers, MA) at room temperature for 2 h. Immunoreactive bands were visualized using an enhanced chemiluminescence detection system (Thermo Scientific, Rockford, IL). Densitometric measurements were done with Image Quant (Molecular Dynamics). GAPDH bands, as a housekeeping protein, were used as an internal control.

### *In vivo* Islet Potency Test

This study was carried out in strict accordance with the Guidelines for the Care and Use of Laboratory Animals of the National Institutes of Health. The protocol was approved by Institutional Animal Care and Use Committee of University of California, Irvine (Permit Number: 2012–3028). Transplantation of human islets to diabetic nude mice was performed as an *in vivo* assessment of islet potency. Diabetic nude mice were anesthetized by isoflurane, and the left kidney was exposed through a lumbar incision. A breach was made in the kidney capsule, and a polyethylene catheter was introduced through the breach and advanced beneath the kidney capsule to generate a subcapsular space. After human islets were cultured with 24 hours culture with (n = 9) or without dh404 (500 nM, n = 8), 2000 IEQ islets were slowly injected through th PE50 tube into subcapsular space. After removing the PE50 tube, the opening was cauterized, and the kidney was repositioned, followed by suturing of muscle and skin. Blood was sampled daily from the tail vein for the determination of blood glucose levels. At 4 weeks after transplantation, islet graft was removed by nephrectomy. Success of transplantation was defined as maintenance of normoglycemia (<200 mg/dl) for at least 3 consecutive days and return of hyperglycemia (>250 mg/dl) after nephrectomy.

### Cytokine and chemokine production from human islet cells

Human islets were cultured with or without dh404 for 24 hours (n = 3, in each group). The supernatant was collected for cytokine and chemokines assay. Analysis was performed using a human cytokine antibody array kit (Ray Biotech, Norcross, GA). Membranes with supernatant were blocked and washed several times followed by the addition of a biotin conjugate anti-cytokine mixture incubated at room temp for 2 hours. Similarly, to the previous cytokine experiment, the addition of conjugated horseradish peroxidase labeled streptavidin (HRP-SA) was incubated at room temp for 2 hours. Membranes were washed and processed with Detection buffer and exposed with X-ray Film (Kodak, Rochester, NY). ImageQuant TL 7.0 (GE healthcare Life Sciences, Pittsburg, PA) was used for analysis [18]. Analysis of cytokines from dh404 treated acini were compared to the non-treated tissue and expressed as a percent of the control.

### Islet Cell Dissociation

Human islets were treated with dh404 (0, 500 nM) for 24hours with or without Hydrogen Peroxide (200 μM, Hydrox Laboratories, Elgin, IL). Approximately 2,000 IEQ human islets were incubated in 1ml accutase solution (Innovative Cell Technologies, Inc, San Diego, CA) at 37°C for 10 min. Accutase was deactivated with cold fetal bovine serum. The accutase-dissociated islet cells were washed with PBS for further assessment [19].

### Analysis of beta cell content by Immunofluorescence

Dissociated islet cells were fixed on glass slides with 2.5% paraformaldehyde (Electron Microscopy Sciences, Washington, PA). Cells were incubated with Universal Blocker Reagent (Biogenex, San Ramon, CA) for 30 min, to reduce nonspecific binding. Thereafter, cells were incubated overnight at 4°C with mouse monoclonal antibody to c-peptide (1:100; Abcam Inc., Cambridge, MA). A secondary antibody was then applied, with goat anti-mouse (1: 200; Alexa Fluor 488 goat anti-mouse IgG). Omission of the primary antibody served as negative control. DAPI was applied to stain cell nuclei. The cellular composition was determined by manually counting the stained cells using a fluorescent microscope. Beta cell content was determined by taking the number of positively stained beta-cells and dividing by the total number of stained cells [19].

### Determination of Human Islet Cell Viability

Dissociated rat islet single cell suspensions were incubated for 30–40 min at 37°C in phosphate-buffered saline (PBS) without Ca^++^ and Mg^++^ with 100 ng/ml of tetramethyrhodamine ethyl ester (TMRE; Life Technologies, Grand Island, NY). TMRE selectively binds to mitochondrial membranes, which allows for the detection of apoptotic cells. After washing with PBS, cell suspensions were stained with 7-aminoactinomycin D (7-AAD; Life Technologies, Grand Island, NY), which binds to DNA when cell membrane permeability is altered after cell death. Cell suspensions were analyzed (minimum 3.0x10^4^ events) using FACScan cytometer with the CellQuest-pro software (Becton, Dickinson and Company) [19]. The islets from 3donors were examined in this study.

### Statistical analysis

Results were expressed as mean ±standard deviation (SD), and the statistical differences between groups were determined by unpaired *t*-test using Excel for Windows software (Microsoft, Redmond, WA). The percentage of cured diabetic nude mice between two groups was determined by Gehan-Breslow-Wilcoxon Test. P values equal to or less than 0.05 were considered significant. In vivo statistics

## Results

### Nrf2 Translocation from cytoplasm to nucleus

**Fig 1:**
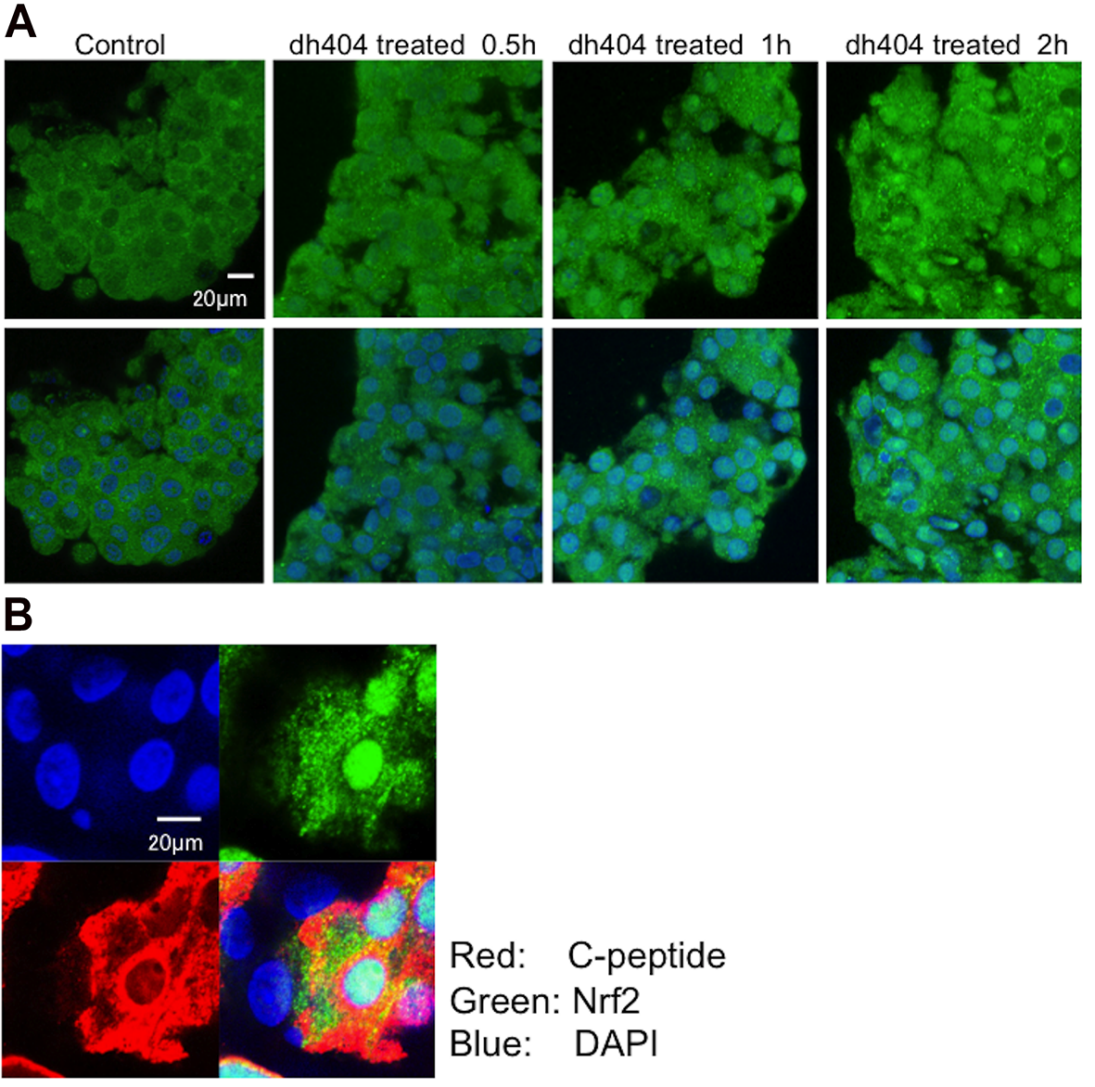
Nrf2 Translocation from cytoplasm to nucleus. **(A)** Human islets were treated with dh404 for 0.5, 1 or 2 hours. The treated and untreated samples were stained with Nrf2 antibody (Green) and DAPI (Blue). The con-focal microscope clearly showed that the Nrf2 translocation from cytoplasm to nucleus in the dh404 treated human islet cells. **(B)** Double staining of Nrf2 and c-peptide antibody demonstrated beta cell specific Nrf2 translocation to nucleus.

Human islets were treated with dh404 (500 nM) or vehicle for 0.5, 1 or 2 hours. The treated and untreated samples were stained with Nrf2 antibody (Green) and DAPI (Blue). The con-focal microscope clearly demonstrated steady increase in translocation of Nrf2 from cytoplasm to nucleus in the dh404-treated human islet cells in a time dependent manner (**Fig 1A**). In addition, the double staining of Nrf2 and c-peptide antibody demonstrated a beta cell specific Nrf2 translocation (**Fig 1B**).

### Effect of treating human islets with dh404 on Nrf2 target gene expression

**Fig 2:**
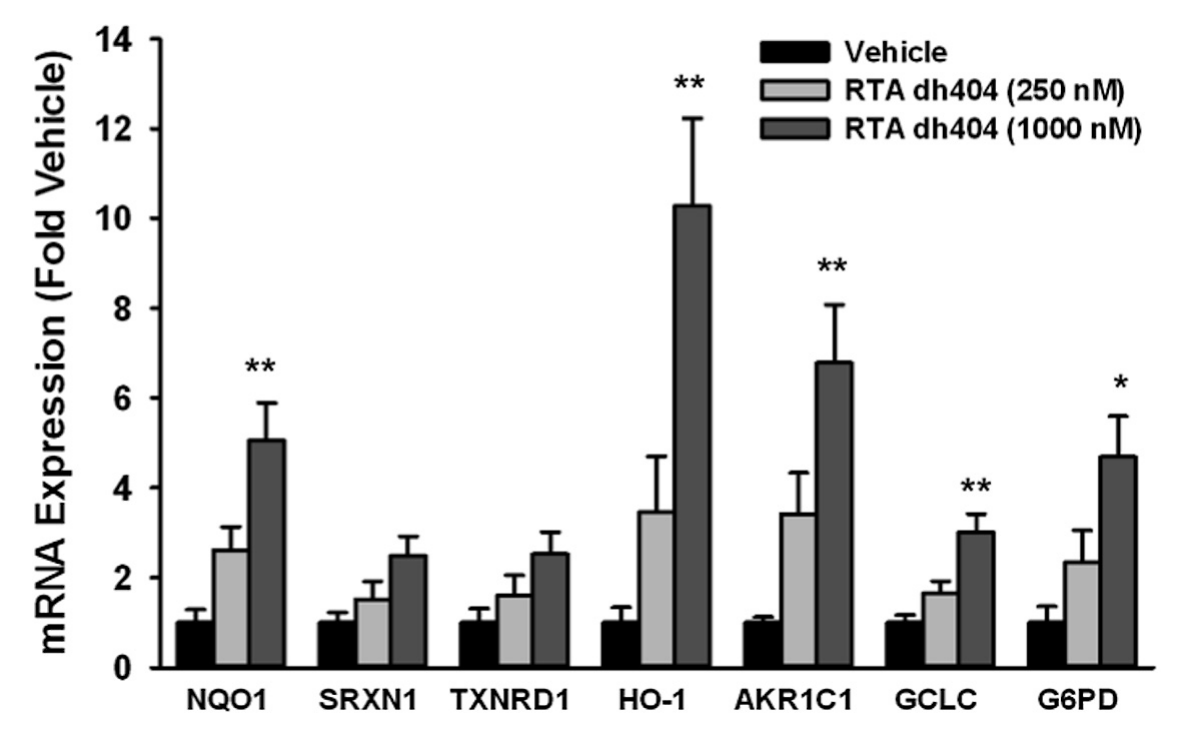
Effect of Treating Human Islets with dh404 on Nrf2 Target Gene Expression. Human islets from three different cadaveric donors were treated with or without dh404 for 12 hours. The target genes of Nrf2 were analyzed by the Quantigene Plex 2.0 assay from Panomics (an Affymetrix company) per manufacturer's protocol. Data were normalized to the housekeeping gene POLR2A and presented as fold vehicle. Data were analyzed by one-way ANOVA followed by Duncan’s post-hoc test with significance set at p <0.05.

Human islets from three different donors were cultured and incubated with dh404 at 250 and 1000 nM in DMSO (0.1% w/v) for 12 hours. Dh404 induced mRNA expression of the Nrf2 targets NAD(P)H:Quinone oxidoreductase (NQO1), Heme-oxygenase-1 (HO-1), aldo-keto reductase family 1 (AKR1C1), Glutamate-cysteine ligase (GCLC), and glucose-6-phosphate dehydrogenase (G6PD) in human islets in a concentration-dependent manner. Dh404 also tended to induce mRNA expression of Sulfiredoxin-1 (SRXN1) and thioredoxin reductase 1 (TXNRD1) (**Fig 2**).

### Enhanced HO-1 expression in dh404 treated human islets

**Fig 3:**
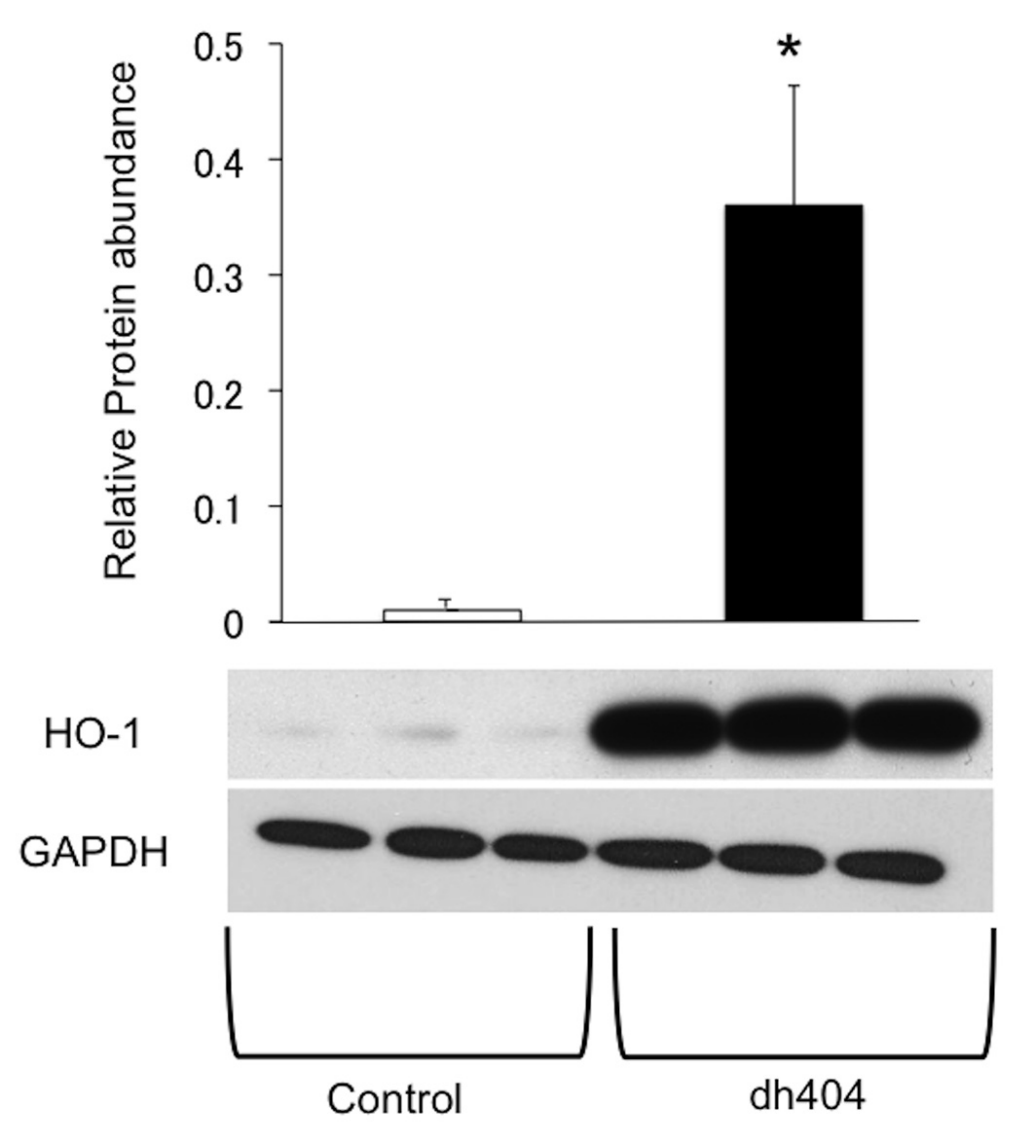
Enhanced HO-1 expression in dh404 treated human islets. Human islets from three different cadaveric donors were treated with or without dh404 500 nM for 24 hours. The Western blot assay was performed. Significant increase in heme oxygenase-1 expression (HO-1) in dh404 treated human islets was observed.

To confirm higher expression of antioxidants induced by dh404 on human islets, human islets from three different cadaveric donors were treated with dh404 1000 nM for 24 hours. The Western blot assay demonstrated significant increase in HO-1 in the treated human islets (**Fig 3**).

### The effect of dh404 on *in vivo* islet potency test

**Fig 4:**
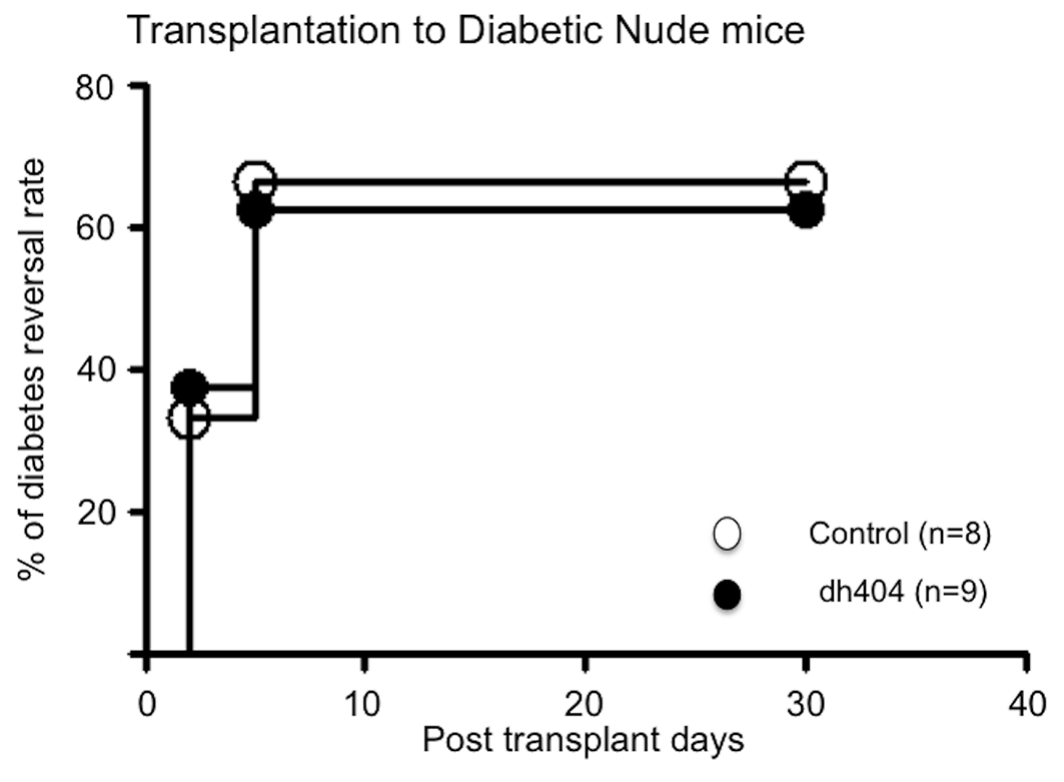
Effect of dh404 on *in vivo* islet potency test. Human islets (2000 IEQ/mouse) treated with (n = 8) or without dh404 (n = 9) were transplanted into the kidney capsule of STZ induced diabetic nude mice to examine islet function in vivo. Dh404 treatment in vitro did not affect islet function after transplant.

To examine the effect of dh404 on islet function in vivo, human islets (2000 IEQ/mouse) treated with or without dh404 were transplanted into the kidney capsule of STZ induced diabetic nude mouse. Dh404 treatment in vitro did not affect islet function in vivo after transplant (**Fig 4**).

### Effects of dh404 on production of inflammatory mediators by human islets

**Fig 5:**
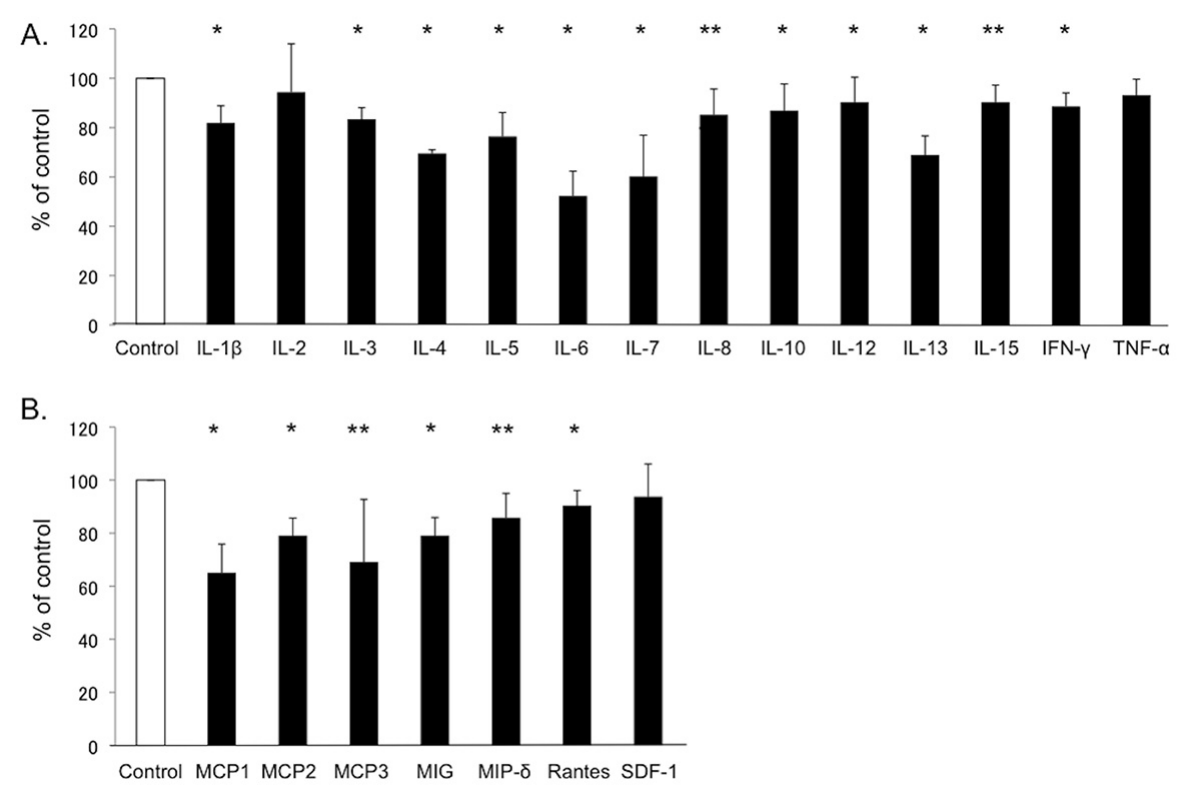
Anti-inflammatory effect of dh404 on human islets. Human islets were cultured for 24 hours with or without dh404 500nM. The concentrations of multiple cytokine/ chemokine were then measured in the supernatant (n = 3, in each). A significant reduction in many inflammatory mediators in the dh404 treated group was observed.

Anti-inflammatory effect is among properties of Nrf2 activator. However, in nude mice, there is no infiltration of inflammatory cells into the transplanted human islets. Human islets are well known to produce many inflammatory mediators, which have negative association with clinical outcome in islet cell transplantation. To investigate the potential inhibitory effects of dh404 on expression of inflammatory mediators, human islets were cultured for 48 hours with or without dh404 (500 nM). The concentrations of multiple cytokine/chemokine were then measured in the supernatant. A significant reduction in many cytokines was observed, including IL-1β, IL-6, IFN-γ and TNF-α, which are well known to have a toxic effect on beta cells (**Fig 5**). Likewise, dh404 significantly reduced MCP-1, a chemokine which has recently been associated with negative clinical outcomes in islet cell transplants.

### Effect of dh404 on beta cell survival under oxidative stress

Under non-stressful condition, the beneficial effects of dh404 on islets were not apparent. Cytoprotective effects of dh404 on human islets against oxidative stress were investigated. HO-1 is well known to have strong cytoprotective effects against oxidative stress on islet cells.

**Fig 6:**
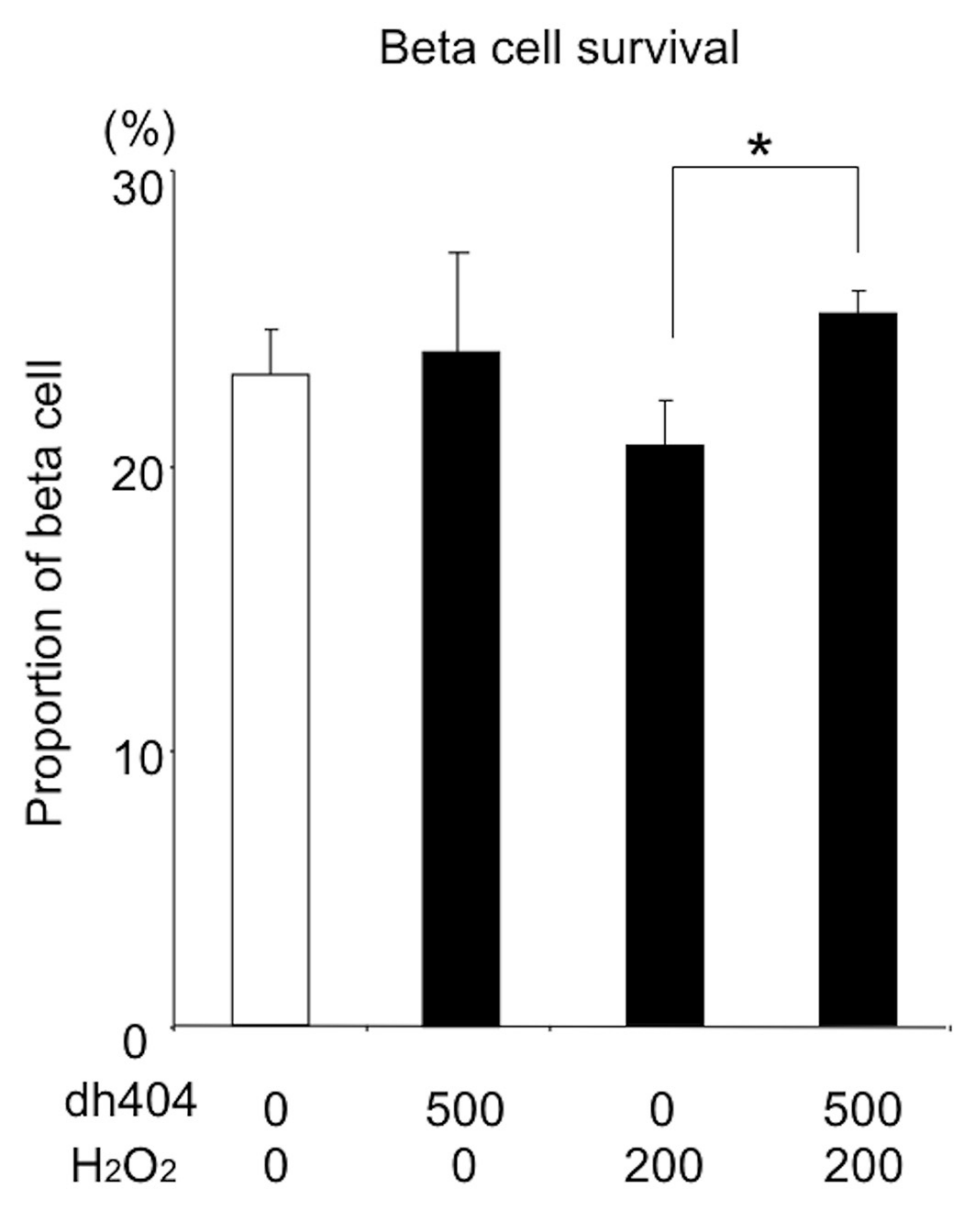
Effect of dh404 on beta cell survival under oxidative stress. Human islets were treated with dh404 500 nM for 24hours with or without H_2_O_2_ 200 μM (n = 3, in each). The islets were dissociated into single cells and stained with C-peptides and DAPI. The proportion of beta cells was significantly higher in the dh404-treated compared to the untreated islets in the presence of H2O2.

The cellular composition in human islets was examined with or without oxidative stress to determine the effect of dh404 on human islet cell survival. Human islets were treated with dh404 (500 nM) for 24hours with or without 200 μM H_2_O_2_. The islets were dissociated into single cells and stained with C-peptide and DAPI. The proportion of beta cells was significantly higher in dh404 treated group (**Fig 6**).

### Cytoprotective effects of dh404 on human beta cells against oxidative stress

**Fig 7:**
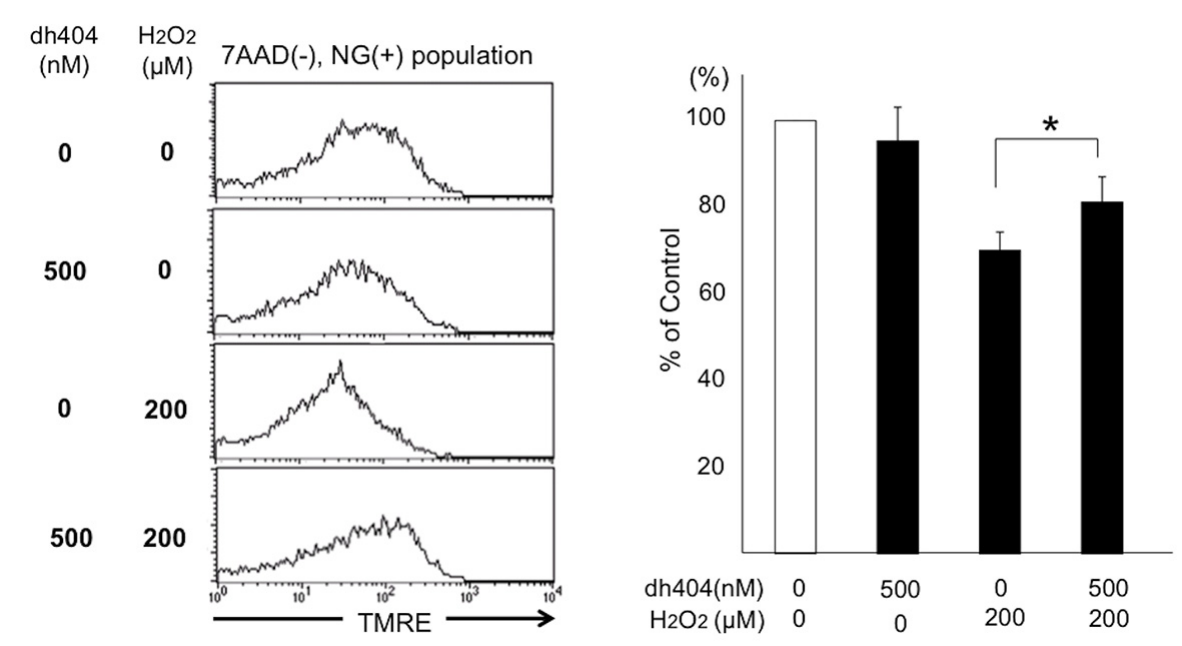
Cytoprotective effects of dh404 on human islets against oxidative stress. Human islets were treated with H_2_O_2_ 200 μM alone or dh404 and H_2_O_2_ 200 μM (n = 3, in each). The percentage of TMRE positive in each group was calculated to evaluate viable beta cells (non-apoptotic). Dh404 significantly protected human beta cells against oxidative stress.

Human islets were treated with or without H_2_O_2_ (200 μM) to determine cytoprotective effects. After human islets were dissociated into single cells, the samples were stained with Newport green, TMRE and 7AAD and analyzed by flowcytometry. Alive beta cell population was identified by 7AAD negative, Newport Green positivity. The percentage of TMRE positive in each group was calculated as viable beta cells. Dh404 significantly decreased H_2_O_2-_ induced beta cell apoptosis (**Fig 7**).

Taken together, Nrf2 activator demonstrated significant cytoprotective effects on beta cells against oxidative stress.

## Discussion

Death of beta cell occurs in patients with both T1DM and type 2 diabetes (T2DM) (although to a much greater extent in T1DM) and it also takes place following the isolation of human islets prior to transplantation [20–22]. Therefore, the identification of mechanisms that may enhance beta cell survival and viability has important therapeutic implications.

In this study, we evaluated the effects of Nrf2 activator, dh404, on human islets. Our data demonstrated cyto-protective and anti-inflammatory effect of pharmacological Nrf2 activation in human beta cells. The treatment of human islets with dh404 significantly increased expression of key anti-oxidant enzymes and conferred protection against oxidative stress in beta cells.

Diabetes mellitus has emerged as an important public health problem with major costs and complications worldwide. In a genetically susceptible person with an environmental trigger such as viruses and toxins, autoimmune destruction of beta cells can occur causing T1DM usually in children and young adults [2]. Oxidative stress is a common feature of both type 1 and type 2 diabetes, and biomarkers of oxidative stress are consistently elevated in the pancreas and other tissues of diabetic patients [23].

Imbalance in production of ROS and antioxidant capacity results in oxidative stress which can lead to cytotoxicity and cell death. Cellular antioxidant enzymes and scavenger molecules are critical in protection against ROS-mediated injury. Pancreatic beta cells are highly vulnerable to oxidative stress which appears to be in part due to the lack of robust antioxidant capacity. In this context Grankvist et al, were the first to demonstrate that in rats alloxan destroys beta cells through a ROS mediated mechanism [24–26]. In another study Lenzen et al, found a significantly lower expression of several key antioxidant enzymes in pancreatic islets as compared to other tissues in normal mice. They found Superoxide Dismutase, Glutathione peroxidase, and Catalase expressions in the pancreatic islets to be 30–40%, 15%, and undetectable, respectively, relative to the values found in the liver [5]. In addition, over-expression of antioxidant enzymes in insulinoma cells results in significantly increased resistance to cytokine-mediated toxicity [27]. To confirm these observations, transgenic mice with the beta cells targeted over-expression of thioredoxin, which is a potent endogeous antioxid and antiapoptotic protein, were shown to be resistant to autoimmune and streptozotocin (STZ)-induced diabetes [28].

Beta cells are exposed to oxidative stress in the processes of islet transplantation such as pancreas preservation, islet isolation, and during and after transplantation. Moreover, in clinical islet transplantation, islets are exposed to a significantly longer ischemic time when compared to other transplant organs. Unlike other transplant organs, transplanted islets' ischemia does not end immediately after transplantation. In fact revascularization has been shown to take as long as 10 days post islet transplantation in the mouse model [29]. Given the role of ischemia reperfusion and prolonged ischemia in production of ROS and tissue damage, the issues outlined above represent a major challenge in islet transplantation.

Nrf2 is a transcription factor that mediates a broad-based set of adaptive responses to intrinsic and extrinsic cellular stresses [30]. Regulation of cellular antioxidant and anti-inflammatory machinery by Nrf2 plays a central part in defense against oxidative stress. Under normal conditions, most of the Nrf2 produced in the cell is held and driven to proteasomal degradation by the repressor molecule Keap1. In the presence of oxidative stress, or an Nrf2 inducer, covalent modifications of cystein residues in the Keap1 molecule disables its ability to bind Nrf2, thereby promoting its translocation to the nucleus [31,32,33]. In the nucleus Nrf2 binds to the antioxidant response element (ARE) on its target genes, promoting the transcription of phase II enzymes including HO-1, glutathione S-transferase A4 (GSTA4), glutathione S-transferase μ1 (GSTM1), NQO1, GCLC, and glutamate cysteine ligase modifier subunit (GCLM) [34,35].

It is notable that diabetes has been found to be one of the many diseases in which Nrf2 mediated expression of endogenous antioxidants is impaired [36]. Several studies have been carried out on animal models which employ Nrf2 inducers to improve rodent islet function. Resveratrol is a naturally-occurring Nrf2 inducer which has been used for centuries as a Chinese herbal medicine. Although, some studies have shown a favorable effect of resveratrol on insulin secretion, others have failed to show any improvement in islet transplanted mice [37] Curcumin, is another naturally occurring Nrf2 inducer the effect of which has been investigated on insulin production in animals' pancreatic islets [38]. Abdel et al reported increase in insulin secretion in curcumin-treated rat islets in a dose dependent manner (6, 8, 10 μmol/L, respectively) [39]. Kanitkar et al reported the protective effect of curcumin on cryopreserved mice islets in terms of islet viability and insulin secretion [40]. They demonstrated higher expression of HO-1 and inhibition of ROS generation in curcumin-treated islets. Likewise, Balamurugan et al. reported the anti-apoptotic effect of curcumin on human islets [41]. The expression of HO-1, GCLM, and NQO1 in islets treated with all tested curcuminoids was significantly higher than un-treated islet.

There appears to be some benefit to using these naturally occurring Nrf2 inducers to improve islets endogenous antioxidant function. Li et al. recently reported that dh404 improved rodent beta cell function not only through antioxidant effects but also autophagic clearance in the setting of diabetes [42]. Our data demonstrated significant increases in mRNA and protein expressions of HO-1 in human islets treated with dh404. HO-1 is a key antioxidant that is known to improve the outcome of islet transplantation [43,44] by decreasing inflammatory mediators and attenuating oxidative stress [45].

The natural antioxidant defense system consists of numerous antioxidant and detoxifying enzymes and substrates which work together to contain oxidative stress and prevent tissue damage. Thus the effects of activation of the endogenous antioxidant system cannot be replicated by administration of one or more exogenous antioxidant compounds. For this reason strategies aimed at enhancing endogenous antioxidant defense systems by restoring Nrf2 activity are more effective in management of oxidative stress as shown in this study.

In this study, we did not observe a significant difference in *in vivo* islet potency test. We recently reported that dh404 pre-treatment improved islet isolation outcome and the diabetes cure rate with transplantation of rat islets to diabetic nude mice [16]. In that study, dh404 was given to the rats prior to islet isolation. Therefore, Nrf2 pathway in islets was already activated during islet isolation. The islet transplantation to diabetic nude mice was immediately performed. In general, the most effective period for anti-oxidative compounds like dh404 for islets is when islets are exposed to strong oxidative or other stress. In the present study, human islets were cultured with dh404 after 24–48 hours post islet isolation. As shown in Figs 6 and 7, dh404 treatment was highly effective in restoring insulin production capacity and viability of beta cells when exposed to oxidative stress. However, in the absence of oxidative stress dh404 had no significant effect on cultured islets’ potency or viability. The effective timing and optimal usage of dh404 analogues for potential use in clinical islet transplantation are presently unclear and requires further investigation.

### In conclusion

Treatment of human islets with the potent synthetic Nrf2 activator, dh404, significantly increased expression of the key anti-oxidant enzymes and decreased expression of inflammatory mediators in pancreatic islets and conferred protection against oxidative stress in beta cells.
